# Cognitive behavioral therapy delivered via digital mobile application for the treatment of type 2 diabetes: Rationale, design, and baseline characteristics of a randomized, controlled trial

**DOI:** 10.1002/clc.23853

**Published:** 2022-07-01

**Authors:** Mario Enrico Canonico, Judith Hsia, Nicole L. Guthrie, Martha Simmons, Prapti Mehta, Paul Lupinacci, Kate Edwards, Kara Mosesso, Michelle Gearhart, Aleksandar Skuban, Marc P. Bonaca, Mark A. Berman

**Affiliations:** ^1^ Department of Medicine, CPC Clinical Research University of Colorado Aurora Colorado USA; ^2^ Department of Medicine University of Colorado Aurora Colorado USA; ^3^ Better Therapeutics Inc. San Francisco California USA

**Keywords:** behavior change, digital therapeutics, type 2 diabetes

## Abstract

**Background:**

The prevalence of type 2 diabetes (T2D) continues to rise in the United States and worldwide. Cognitive behavioral therapy (CBT) has been shown to improve glycemic control in patients with T2D, but broad implementation has been limited by inherent access and resource constraints. Digital therapeutics have the potential to overcome these obstacles.

**Hypothesis:**

To describe the rationale and design of a trial evaluating the efficacy and safety of a digital therapeutic providing CBT to improve glycemic control in adults with T2D.

**Methods:**

This randomized, controlled, multicenter, Phase 3 trial evaluates the hypothesis that BT‐001, an investigational digital therapeutic intended to help patients with T2D improve their glycemic control, on top of standard of care therapy, will lower hemoglobin A1c (HbA1c) compared to a control app across a broad range of patients in a real‐world setting. The study is designed to provide evidence to support FDA review of this device as a digital therapeutic. The intervention is provided within the digital application (app) and includes no person‐to‐person coaching. The primary endpoint is the difference in HbA1c change from baseline to 90 days for BT‐001‐allocated subjects compared with those assigned to the control app. Safety assessment includes adverse events and adverse device effects. The study incorporates pragmatic features including entirely remote conduct with at‐home visits for physical measures and blood sample collection.

**Conclusions:**

This randomized, controlled trial evaluates a cognitive behavioral intervention delivered via smartphone app which has the potential to provide a scalable treatment option for patients with T2D.

**Abbreviations**
App: application
BP: blood pressure
CBT: cognitive behavior therapy
HbA1c: hemoglobin A1c
nCBT: nutritional cognitive behavioral therapy
PRO: patient‐reported outcomes
SD: standard deviation
SOC: standard of care
T2D: type 2 diabetes

## 1 INTRODUCTION

Over 28 million Americans have diagnosed, and a further eight million have undiagnosed, type 2 diabetes (T2D).^1^ Despite the introduction of new pharmacological agents and endorsement of early combination therapy,^2^ only half of patients with diabetes in the United States achieve hemoglobin A1c (HbA1c) <7%.^3^

In‐person cognitive behavioral therapy (CBT)‐based interventions have been shown to improve glycemic control in patients with diabetes. In a meta‐analysis of 22 randomized trials including 2619 subjects with type 1 or T2D, CBT‐based interventions lowered HbA1c by 0.275% (95% confidence interval [CI]: −0.443 to −0.107; *p* < .01).^4^ In another meta‐analysis of 20 other randomized trials including 2900 patients with T2D, CBT was more beneficial than the control condition with a standardized mean difference in HbA1c of −0.97 (95% CI: −1.37 to −0.57), corresponding to an effect size of −1.56% in absolute units.^5^ In these trials, interventions were provided face‐to‐face, by telephone or internet, in one‐on‐one, peer‐to‐peer, or small group formats. Despite promising data, the imbalance between the numbers of patients with diabetes and numbers of trained cognitive behavioral therapists precludes widespread implementation of this therapeutic approach.

Digital therapeutics that deliver behavioral interventions have the potential to increase access since they are inherently scalable and more broadly accessible to patients including rural residents, those lacking transportation, childcare or time off work to attend appointments.^6^ BT‐001 is a digital therapeutic application (app) providing CBT via smartphone supporting dietary change, physical activity, and medication adherence. An earlier version of the app, when paired with health coaching, was associated with a 1.1% fall in HbA1c after 3 months in a sample of 69 adults with T2D^7^; in a separate pilot study (*n* = 74), fasting blood glucose fell by 23 mg/dl after about 3 months.^8^ Herein, we describe the design of a randomized, controlled trial assessing the efficacy and safety of this digital therapeutic app that delivers CBT with the intent to improve glycemic control in adults with T2D.

## 2 METHODS

### 2.1 Study design

**Figure 1:**
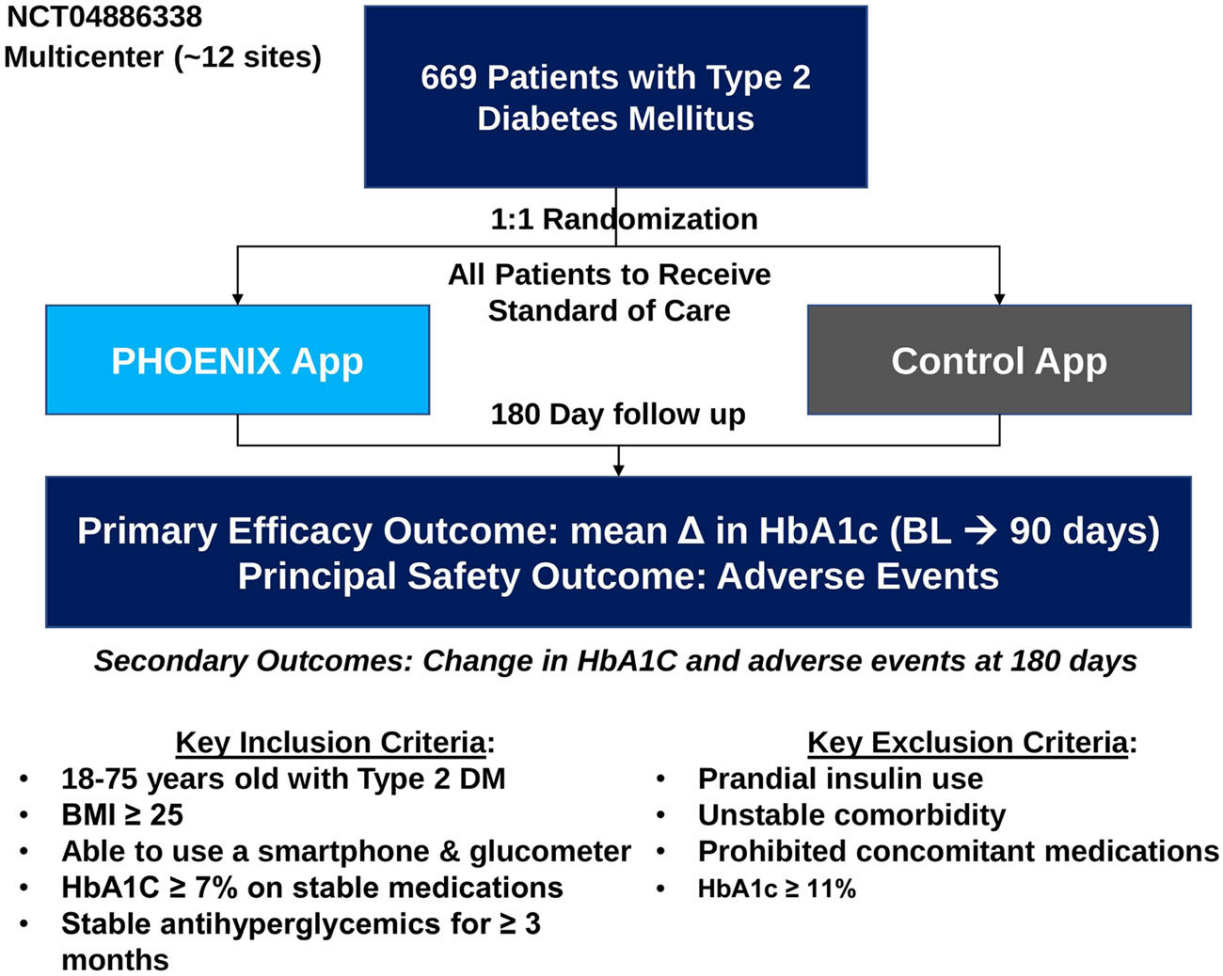
Study schema. BL, baseline; BMI, body mass index; DM, diabetes mellitus; HbA1c, hemoglobin A1c.

Approximately 650 adults with T2D were to be randomized (1:1) to receive access to BT‐001 (called PHOENIX in the app) or to a control app, both on top of standard of care (SOC) for 180 days (Figure 1). Electronic informed consent was obtained consistent with Institutional Review Board requirements. Inclusion and exclusion criteria (Table 1) were chosen to enroll a representative population of adult outpatients with T2D across the United States. Eligible subjects had HbA1c ≥7.0% a stable antidiabetic regimen for the prior 4 months and access to a smartphone. Major exclusion criteria included current use of prandial insulin and current smoking.

**Table 1:** Inclusion and exclusion criteria

Inclusion	Age 18–75 years
	Type 2 diabetes
	Body mass index ≥25 kg/m^2^
	Possesses a smartphone (iPhone or Android only) capable of running the app
	No change in antidiabetic medications in the 4 months before randomization
	HbA1c level ≥7%
	Willing to use an FDA approved glucometer for self‐monitoring blood glucose
	Capable of providing informed consent
Exclusion	Unable to understand, consent to, or comply with the study protocol for any reason, including the inability to read or comprehend English
	Current prandial insulin
	Active eating disorder
	Taking or planning to take oral steroids, chemotherapy, weight loss medications, atypical antipsychotic medications (e.g., risperidone [Risperdal®], olanzapine [Zyprexa®], quetiapine [Seroquel®], ziprasidone [Geodon®], aripiprazole [Abilify®], paliperidone [Invega®], lurasidone [Latuda®])
	Change in antidepressant or antianxiety medication within the past 3 months
	History of or planned bariatric surgery
	Current use of marijuana, cocaine, opioid painkillers, or other addictive substances
	Current use of tobacco products or use of tobacco products within the past 6 months;
	Consumption of alcohol above defined thresholds: For women: more than three drinks in a single day, or more than seven drinks per week; and for men: more than 4 drinks in a single day, or more than 14 drinks per week
	Unstable or life‐threatening medical illness, precluding full compliance with the study protocol
	Unresolved, presumed, or confirmed COVID‐19 diagnosis
	For women only: pregnant (or lactating) or having the intention of becoming pregnant during the time frame of the study
	Concurrent enrollment in any other clinical trial
	Considered unreliable by the investigator, or having any condition which, in the opinion of the investigator, would not allow safe participation in the study
	HbA1c ≥ 11%

Abbreviation: HbA1c, hemoglobin A1c.

Subjects in both treatment groups were directed to download an app delivering ongoing study surveys; for those in the BT‐001 group the app also delivers the behavioral intervention. Randomized subjects who did not complete the onboarding process directed by their assigned app within 2 weeks were replaced.

Investigators can adjust antihyperglycemic medications as needed consistent with SOC during the study period and all concomitant therapies are permitted.

ClinicalTrials. gov number for this study is NCT04886388.

### 2.2 Study endpoints

The primary efficacy endpoint is the treatment group difference in the mean change in HbA1c from baseline to Day 90. The secondary efficacy endpoint is the change from baseline to Day 180. Exploratory endpoints include physical measures, biomarkers, healthcare utilization, and patient‐reported outcomes (PRO). The latter include the 12‐item Short Form health survey (SF‐12), a validated measure of physical and mental health^9^ and the Patient Health Questionnaire‐9 (PHQ‐9), which provisionally identifies individuals with depression.^10^ Participants with scores suggestive of severe depression and/or suicidality will be withdrawn from the study and directed toward appropriate medical care. BT‐001‐allocated subjects also complete the Net Promoter Score,^11^ an assessment of user experience with the app. A complete list of study endpoints is provided in Table 2.

**Table 2:** Efficacy and safety endpoints

Efficacy endpoints	
Primary	Difference in the mean change from baseline in HbA1c at Day 90 between the treatment groups
Secondary	Difference in the mean change from baseline in HbA1c at Day 180 between the treatment groups
Exploratory	The difference in the mean changes from baseline in outcomes related to blood glucose control (HOMA2‐IR, fasting insulin, fasting blood glucose) at 90 and 180 days between groups The difference in the mean changes from baseline in outcomes related to blood lipids (LDL‐, HDL‐, and total cholesterol, triglycerides; small, medium, and total LDL particle numbers; LDL peak size and the associated LDL pattern; large HDL particle number; apolipoprotein A1, apolipoprotein B) at 90 and 180 days between groups The difference in the mean changes from baseline in outcomes related to inflammation (high sensitivity C‐reactive protein) at 90 and 180 days between groups The difference in the mean changes from baseline in BP (systolic and diastolic) and cardiovascular risk score at 90 and 180 days between groups Mean changes from baseline in the count and dose of antihyperglycemic and antihypertensive medications at 90 and 180 days within each group The difference in the mean changes in outcomes related to weight change (weight change, percent body weight loss, and body mass index) at 90 and 180 days between groups The difference in the mean changes in PRO scores at 90 and 180 days between groups Net Promoter Score at 90 and 180 days within the BT‐001 group
Safety endpoints	Occurrence, relatedness, and severity of adverse events and adverse device effects at Days 90 and 180

Abbreviations: BP, blood pressure; HbA1c, hemoglobin A1c; HDL, high density lipoprotein; HOMA2‐IR, homeostasis model assessment 1‐insulin resistance; LDL, low density lipoprotein; PRO, patient‐reported outcomes.

### 2.3 BT‐001 app

The BT‐001 app contains a novel behavioral intervention, nutritional CBT (nCBT), which incorporates techniques to:

(1) identify maladaptive thoughts based on misinformed or false underlying core beliefs (e.g., those related to macronutrient fears, the hedonic nature of eating, physical exertion, other perceived barriers to changing lifestyle) that lead to disease‐promoting behaviors;

(2) replace these maladaptive core beliefs and thought patterns with adaptive ways of thinking developed from rational reflection;

(3) provide collaborative (between participant and device) construction of behavioral exercises to test core beliefs;

(4) leverage additional validated behavioral techniques to enhance a participant's capacity to solve problems, plan activities, and navigate interfering emotions or thoughts.

Within the app, participants initially view short videos outlining the goals and features of behavioral therapy and can access answers to frequently asked questions. Key features in the app are then introduced through a required first‐time user experience before being given full access. The app helps patients understand the steps they should prioritize by presenting them with a treatment plan which summarizes their daily and weekly goals; this plan is essentially their home screen.

A key goal is to complete one nCBT lesson module and at least one skill module each week. All patients are exposed to the same course of lesson modules, each of which is expected to take 10–20 min to complete and addresses core beliefs in one or more of the areas in Table 3. Skill modules are behavioral exercises related to the lesson, often involving activities outside the app. Each lesson explains the rationale and benefits of the skill exercise in reference to the core topic being explored. The skill exercises provide an opportunity for participants to practice skills related to behavioral beliefs. The method by which participants must practice these skills is designed to enhance executive function tasks such as planning, problem solving, and goal setting.

**Table 3:** Core beliefs addressed in BT‐001 lessons

Core beliefs	Examples
	Beliefs about
Beliefs about health and disease	The ability to modify severity of type 2 diabetes with behavioral changes
	The contribution of genetics and environmental factors to development of type 2 diabetes
Personal beliefs and barriers	Capacity for incremental change in personal behavior
	Individual evolution of personal identity over time
Beliefs about nutrition and the importance of various food types	Different types of food high in carbohydrate and their impact on health
	The necessity of animal protein as the core element of meals
Hedonic‐related beliefs about pleasant or unpleasant sensations experienced by eating or exercising	The amount of effort required for exercise
	The ability of food preferences to change after exposures to new food types
Beliefs about exercise	The role of exercise in relationship to dietary change for health improvement and weight loss
	The role of strength training in improving type 2 diabetes
Beliefs about individual interaction with family, culture, and society	The importance of personal health in relationship to family obligations
	The role of social connections in physical health

The behavioral activation component of nCBT also reinforces adaptive behaviors practiced in the skill modules by encouraging daily self‐reporting of plant‐based meals consumed (the large majority of the meal consisting of whole or minimally processed plant foods), minutes of exercise (moderately intense aerobic exercise and strength training consistent with clinical guidelines), medication adherence, and biometrics (fasting blood glucose, weight and blood pressure (BP) if hypertensive). The app sets expectations with patients about therapeutic levels of behavior change in nutrition and exercise, while also recognizing inherent individual variability. Weekly behavioral goals are advanced using an algorithm based on recent self‐reported behavior. The intent of the algorithm is to advance changes in a manner that will maintain or increase self‐efficacy, however, patients can increase or decrease the goal suggested by the algorithm. Behavioral levels are reinforced during weekly goal‐setting, enabling patients to see their weekly goal in relation to longer term therapeutic targets.

The app applies safety‐by‐design. Risks of hypoglycemia and hypotension are mitigated through self‐monitoring and patient‐facing biometric notifications such as alerts based on self‐reported glucometer results. BT‐001 is intended for use between clinic visits within 90‐day treatment cycles, each of which includes 13 n‐CBT lessons. The n‐CBT content for the second treatment cycle is available to patients during the first treatment cycle if they choose to work ahead. While nCBT is a time‐limited therapy, BT‐001 is designed to be repeated for additional treatment periods with new lessons if necessary.

### 2.4 Study procedures

The study collects data on concomitant medications, physical measures, and laboratory data including HbA1c, lipids, glucose, insulin, and high sensitivity C‐reactive protein. Adverse device effects and adverse events are identified through patient questionnaires, contacts with study staff, and the product support team (Better Therapeutics Helpdesk).

### 2.5 Statistical considerations

The primary efficacy endpoint, treatment group difference in HbA1c change from baseline to Day 90, will be assessed using an analysis of covariance with main effect of treatment and baseline HbA1c as a covariate in the modified intention‐to‐treat population which includes all randomized subjects who complete onboarding for their assigned app and who had both a baseline and Day 90 HbA1c assessment. If there are statistically significant key baseline or demographic characteristics, these factors will be incorporated into the statistical model as covariates. Adverse events and adverse device effects will be summarized.

The sample size estimate assumed a standard deviation (SD) of 1.4% for HbA1c change from baseline based on previous studies conducted by the sponsor, a common SD for the two randomized treatment groups, and a 0.4% difference between treatment groups in HbA1c change from baseline. A nominal sample size of 259 participants per treatment group would provide 90% power for a two‐sided *t*‐test (*α* = 0.05); a sample size of 324 participants per group was planned to allow for 20% attrition.

### 2.6 Study organization

The trial is sponsored by Better Therapeutics Inc. (San Francisco, CA) and analyzed in partnership with CPC Clinical Research (Aurora, CO), a nonprofit Academic Research Organization affiliated with the University of Colorado. An Executive Committee which provides study oversight will have unrestricted access to the necessary data to submit the study results for publication.

## 3 RESULTS

### 3.1 Baseline characteristics of study population

From April 22 to November 17, 2021, 12 sites in the United States randomized 725 subjects of whom 669 completed onboarding for their assigned app. Mean age at baseline was 58 ± 9 years (mean ± SD), 56% were female, 28% Black or African American, and 4% Asian; 16% were Latino (Table 4). Median neighborhood household income was $69 000 and 40% of subjects reported not having a college degree. Participants reported a mean of 11 ± 8 years since diagnosis and 3 ± 2 comorbidities in addition to T2D and overweight or obesity. At baseline, 68% were using two or more antihyperglycemic medications, 69% were using pharmacotherapy for hypertension, and 64% were on pharmacotherapy for hyperlipidemia. Cardiac disorders at baseline were reported by 41 subjects (6%).

**Table 4:** Baseline characteristics

	Overall cohort (*n* = 669)
Age, years, mean (SD)	58 (9)
Female, *n* (%)	376 (56)
Race, *n* (%)	
White	400 (60)
Black or African American	190 (28)
Asian	30 (4)
American Indian or Alaskan Native	7 (1)
Native Hawaiian or other Pacific Islander	2 (0.3)
Other (includes multiple races)	40 (6)
Ethnicity, *n* (%)	
Hispanic or Latino	105 (16)
Non‐Hispanic or Latino	564 (84)
Body mass index, mean (SD)	35 (7)
Educational level, *n* (%)	
High school degree or less	82 (12)
Some college or college degree	470 (70)
Graduate degree	117 (18)

## 4 DISCUSSION

This randomized, controlled trial evaluates the efficacy and safety of CBT delivered via digital mobile app intended to improve glycemic control among patients with T2D. Key advantages of the digital app over in‐person CBT include its scalability, standardization of the intervention, avoidance of stigma associated with mental healthcare and ease of access. The latter is particularly important for rural residents and patients with time, mobility or transportation limitations.

The study design includes several pragmatic aspects reflecting constraints associated with the COVID‐19 pandemic, including entirely remote conduct with electronic informed consent as well as blood sample collection and physical measurements performed by mobile phlebotomy units conducting home visits. Subjects allocated to the intervention group are not required to use the mobile app with any specific frequency or for any specific duration. Data collected via smartphone include PRO for both the intervention and control groups. In addition, subjects in the intervention group input weight, BP, glucose values and self‐reported dietary, and physical activity data directly into the app. These activities engage the subjects in self‐management and reinforce the close relationship smartphone owners have with their devices.^12^ The app also collects engagement data such as duration of app use, number and timing of lessons and skill exercises completed.

Limitations of the study include the current availability of BT‐001 only in English and the requirement for an Android or Apple smartphone which may exclude some segments of the otherwise eligible population. In addition, pregnant women were excluded due to the impact of pregnancy on HbA1c as were smokers due to the potential impact of smoking cessation on cardiometabolic parameters. Strengths of the study include its randomized, controlled design, conduct in a real world setting, and broad eligibility criteria increasing the generalizability of its findings. The impact of this approach is reflected in the diverse racial/ethnic minority representation in the study.

In‐person CBT has been shown to improve medication adherence and lower HbA1c. Given the disconnect between the numbers of patients with T2D and trained therapists, widespread provision of CBT is impractical. If this intervention delivered via digital app proves effective at lowering HbA1c, it may provide an additional therapeutic option for adults with T2D.

## CONFLICTS OF INTEREST

N. L. Guthrie, M. Simmons, P. Mehta, P. Lupinacci, K. Edwards, K. Mosesso, M. Gearhart, A. Skuban, and M. P. Bonaca are employees of Better Therapeutics and own stock in the company. Drs. M. E. Canonico, M. P. Bonaca, and J. Hsia receive salary support from CPC, a nonprofit academic research organization affiliated with the University of Colorado, that receives research grant/consulting funding from: Abbott, Agios, Alexion Pharma, Alnylam, Amgen, Angionetics, ARCA Biopharma, Array, AstraZeneca, Atentiv, Audentes, Bayer, Better Therapeutics, Brigham and Women's Hospital, Bristol‐Myers Squibb, Cardiol Therapeutics, CellResearch, Cook Medical, Cook, CSL Behring, Eidos Therapeutics, EP Trading Co., Esperion Therapeutics, EverlyWell, Faraday, Fortress Biotech, HDL Therapeutics, Heartflow, Hummingbird Bioscience, Insmed, Janssen, Kowa Research, Lexicon, Merck, Medtronic, Moderna, Novate Medical, NovoNordisk, Pfizer, PhaseBio, PPD Development, Prairie Education and Research, Prothena Biosciences, Regeneron, Regio Biosciences, Sanifit Therapeutics, Sanofi, Smith and Nephew, Stealth BioTherapeutics, University of Colorado, University of Pittsburgh, Worldwide Clinical Trials, Wraser, Yale Cardiovascular Research Group. Dr. J. Hsia also reports owning AstraZeneca stock and Dr. M. P. Bonaca reports salary support from an AHA SFRN under award numbers 18SFRN3390085 (BWH‐DH SFRN Center) and 18SFRN33960262 (BWH‐DH Clinical Project). Dr. Bonaca also reports stock in Medtronic and Pfizer and consulting fees from Audentes.

## Data Availability

The data that support the findings of this study are available from the corresponding author upon reasonable request.
